# Flow Cytometry in the Diagnosis of Canine B-Cell Lymphoma

**DOI:** 10.3389/fvets.2021.600986

**Published:** 2021-03-19

**Authors:** Fulvio Riondato, Stefano Comazzi

**Affiliations:** ^1^Dipartimento di Scienze Veterinarie, Università degli Studi di Torino, Grugliasco, Italy; ^2^Dipartimento di Medicina Veterinaria, Università degli Studi di Milano, Lodi, Italy

**Keywords:** dog, lymphoma, B-cell, flow cytometry, diagnosis, prognosis

## Abstract

B cell lymphoma (BCL) is a heterogeneous group of lymphoid malignancies which comprise the majority of canine lymphomas. Diffuse large B cell lymphoma is the most common lymphoma subtype in dogs but other subtypes (e.g., marginal zone lymphoma, follicular lymphoma, mantle cell lymphoma, and others) have been described. This review aims to explore the use of flow cytometry to refine the diagnosis of canine BCL. Particular emphasis will be given to the possible identification of peculiar immunotypes, putative prognostic markers, staging and minimal residual disease.

## Introduction

Flow cytometry (FC) of fine needle aspirates has been increasingly applied as first-line analysis in cases of suspected lymphoma in dogs. It is mainly used to investigate the immunophenotype of the cells and refine the diagnosis obtained from other morphological diagnostic techniques such as cytology and histopathology. In addition to the cell lineage definition, recent studies investigated flow cytometric parameters with a potential clinical and diagnostic relevance. The present review is aimed to summarize these aspects in B-cell lymphoma (BCL).

## Identification Of B-Cell Lineage

Current histologic and cytologic classification of lymphomas (WHO and Kiel updated, respectively) (1, 2) requires the identification of the lineage of the cells. A possible correlation between updated Kiel classification and WHO classification for BCLs according to (3) is reported in Table 1. FC is one of the best available methods to define the immunophenotype of discrete cells in routine diagnostics. The major advantages include the multiparametric approach and the availability of a wider panel of antibodies compared to other techniques. This allows the description of the antigenic pattern of cells and provides information about the lineage, differentiation/maturation, activation, and other specific features. Unfortunately, only a few antibodies are commercially available for the identification of B-cells in dogs. Common antibodies used to characterize B-cells and their expected immunoreactivity in non-neoplastic cells are reported in Table 2. The most widely used B-cell markers in FC are IgM, CD21, CD22, and CD79. Antibodies directly conjugated with a fluorochrome are preferred in routine diagnostics; they allow a more effective multicolor approach and a faster labeling protocol. Similarly, the staining procedure for membrane epitopes (e.g., CD21, CD22, and IgM) is faster and better preserves the morphology of the cells. In contrast, the detection of the transmembrane antigen CD79 requires an additional cell permeabilization step to recognize the intracellular epitope. Monoclonal antibodies against the alpha and beta heterodimers (CD79a and CD79b, respectively) have been developed and, to the authors' experience, they provide similar results. The combined evaluation of different markers may provide information on the maturation stage. For instance, CD79 is detected in all stages of B-cell development, IgM is expressed starting from an immature B-cell stage while CD21 and CD22 are present only in mature B-lymphocytes. Although currently not available on the market, some monoclonal antibodies for canine CD20 have been developed and validated in FC on clinical samples (4–6). Similar to CD21 and CD22, CD20 is expressed by mature B cells, and may be of aid in cases of ambiguous results with the former markers. Other non-specific markers for B-cells may be added to FC panels to discriminate their activation or maturation status. Among these, the pan-leukocytic markers CD45 and CD18 are generally expressed with lower intensity in B-cells than in other leukocytes (7). CD25 is generally expressed by activated B-cells and MHC II is expressed by mature B-cells. In humans, plasma cells are CD38+, they retain CD79 but lose CD21 and IgM expression. To date, no specific canine plasma cell marker is commercially available. Further, there are no consistent data about the expression of the commonly used B-cell markers on canine plasma cells.

**Table 1:** Possible correlation between updated Kiel classification and WHO classification for B-Cell Lymphomas according to (3).

**Updated Kiel classification**		**WHO classification**
Low-grade BCLs	Small B-cell lymphoma	
	Small lymphocytic	B-cell CLL/small lymphocytic lymphoma? OR low-grade small BCL NOS?
	Prolymphocytic	
	Lymphoplasmacytic	Lymphoplasmacytic lymphoma
	Marginal zone	Nodal MZL, extranodal MZL, splenic MZL
	Centroblasto-centrocytic	Follicular lymphoma grade I/II
High-grade BCLs	Centroblastic monomorphic, follicular subtype	Follicular lymphoma grade III
	Centroblastic monomorphic, diffuse subtype	DLBCL
	Centroblastic polymorphic	DLBCL
	Immunoblastic	DLBCL
	Anaplastic/mediastinal	Mediastinal BCL (DLBCL)
	Burkitt type	Burkitt lymphoma
	Plasmacytoid	–
	Small cell NOS	Mantle cell lymphoma?

*BCL, B-cell lymphoma; CLL, chronic lymphocytic leukemia; DLBCL, diffuse large B-cell lymphoma; MZL, marginal zone lymphoma; NOS, not otherwise specified*.

**Table 2:** Common antibodies used for the characterization of B-cells in dogs and expected reactivities.

**Target**	**Antibody clones**	**Reactivity**
CD18	CA1.4E9	All leukocytes
CD19	4E9	B-cells, including early precursors
CD20	NCD1.2	Mature B-Lymphocytes
	1E4	
	6C8	
CD21	CA2.1D6	Mature B-lymphocytes
CD22	RFB4	Mature B-lymphocytes
CD25	P4A10	Activated lymphocytes
CD34	1H6	Precursors
CD45	YKIX716.13	All leukocytes
	CA12.10C12	
CD44	IM7	All hematopoietic cells
CD79a(cy)	HM57	B-cells, including early precursors
CD79b(cy)	AT107-2	B-cells, including early precursors
IgM	polyclonal	Immature/Mature B-cells
MHC II	YKIX334.2	Monocytes, Histiocytes, Lymphocytes
	CA2.1C12	
Ki67 (nuclear)	MIB-1	Proliferating cells

*cy, cytoplasmic*.

## Differentiating Reactive And Neoplastic B-Cells

A restricted expression of kappa or lambda light chain is consistent with a B-cell clonal expansion and the evaluation of the kappa/lambda ratio is the reference flow cytometric method to diagnose a neoplastic proliferation of B-cells in humans. Unfortunately, the kappa/lambda ratio is very low in the normal canine lymphoid populations (8) with lambda chains strongly exceeding the kappa ones. Also, the majority of canine BCL (19/23) showed a restricted expression of lambda chain in a study using immunohistochemistry (1). Therefore, although technically feasible, the kappa/lambda ratio has a poor diagnostic significance to distinguish reactive and neoplastic B-cell expansions in dogs.

In dogs, B cells generally represent a minority of the population in non-neoplastic lymph nodes (9, 10), thus an expansion of B cells suggests a possible BCL. However, there is no consensus about a cutoff value to differentiate BCL and a reactive proliferation in lymph nodes and variable criteria have been adopted in different studies. In two studies, the mean percentage of CD21+ cells in lymph node aspirates from clinically normal dogs was 31.4% (95%, CI 24.7–38%) (11) and 33.9% (+/− 11.8%) (12), respectively. Other studies adopted cutoff values of 60% (9, 13) and 65% (14) to define a B-cell lineage in neoplastic lymph nodes. However, a high percentage of residual non-neoplastic T lymphocytes is often found in BCLs, and setting such high cutoff values may significantly increase the rate of false non-neoplastic results.

**Figure 1:**
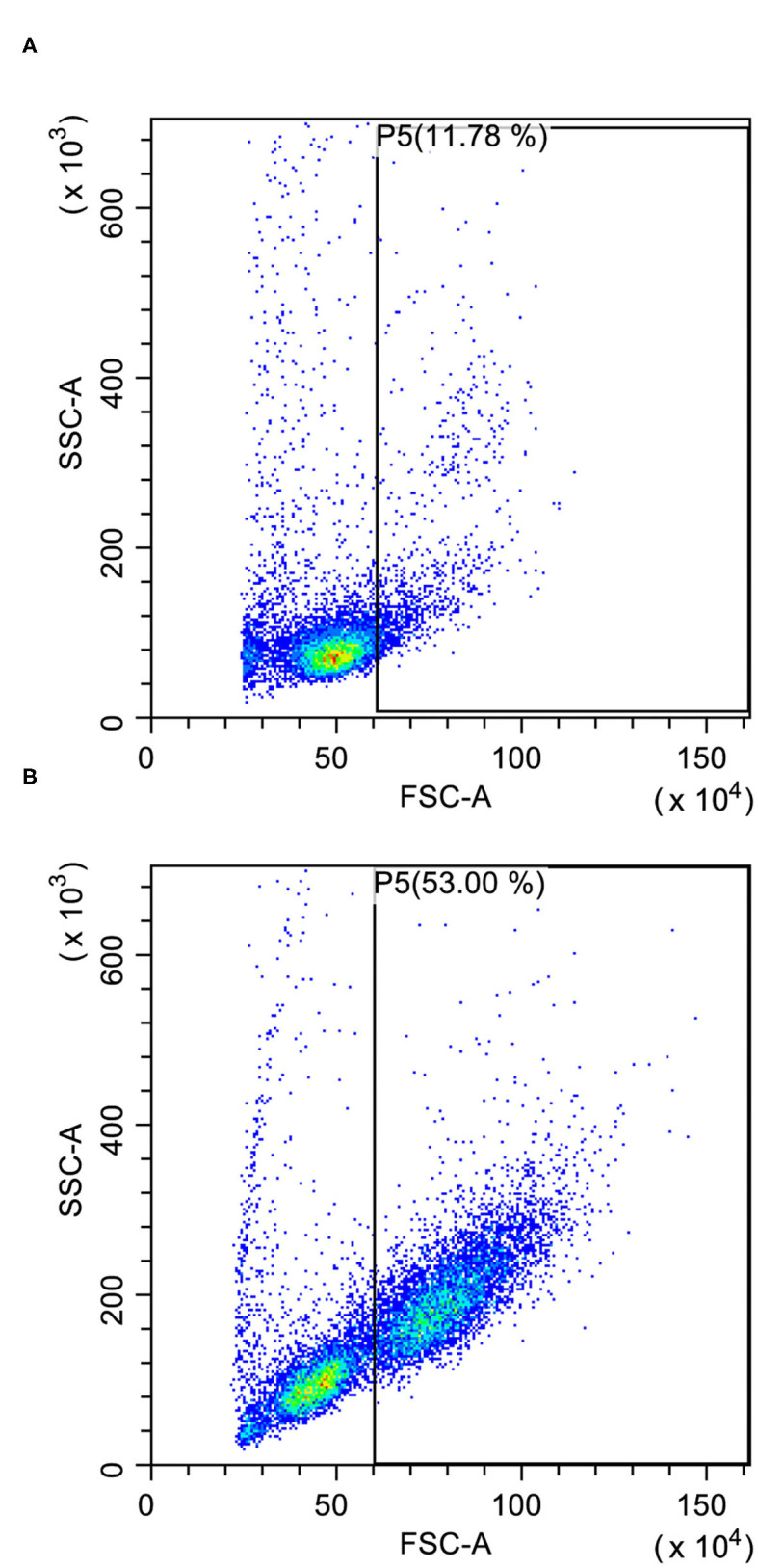
Forward scatter (FSC) vs. side scatter (SSC) plots after doublet exclusion. **(A)** Reactive lymph node with few large-sized cells (P5); some granulocytes are recognizable in the gate. **(B)** Large B-cell lymphoma with many large-sized cells (P5) easily recognizable even in the presence of a substantial residual population of small lymphocytes.

The evaluation of cell size in the lymph node aspirate may also help to support the diagnosis of lymphoma since lymphocytes are mostly small-sized in both normal and reactive lymph nodes (11) (Figure 1). Cell size in FC is measured through Forward Scatter (FSC) properties. FSC has a good correlation with the size of the cell in cytology but not with the size of the nucleus, which is the common morphologic feature used to describe neoplastic cells as small, medium, or large. Coupling CD21 expression with FSC greatly improves the ability of FC to differentiate BCLs from reactive lymph nodes. In a recent study, on different BCLs, most lymphoma cases exhibited an FSC higher than 469 U, a value similar to FSC of residual T lymphocytes and higher than FSC of normal B lymphocytes. Moreover, a median FSC higher than 720 U (corresponding to about 1.6 times the one of residual T lymphocytes) was adopted to define large-sized cases (15).

**Figure 2:**
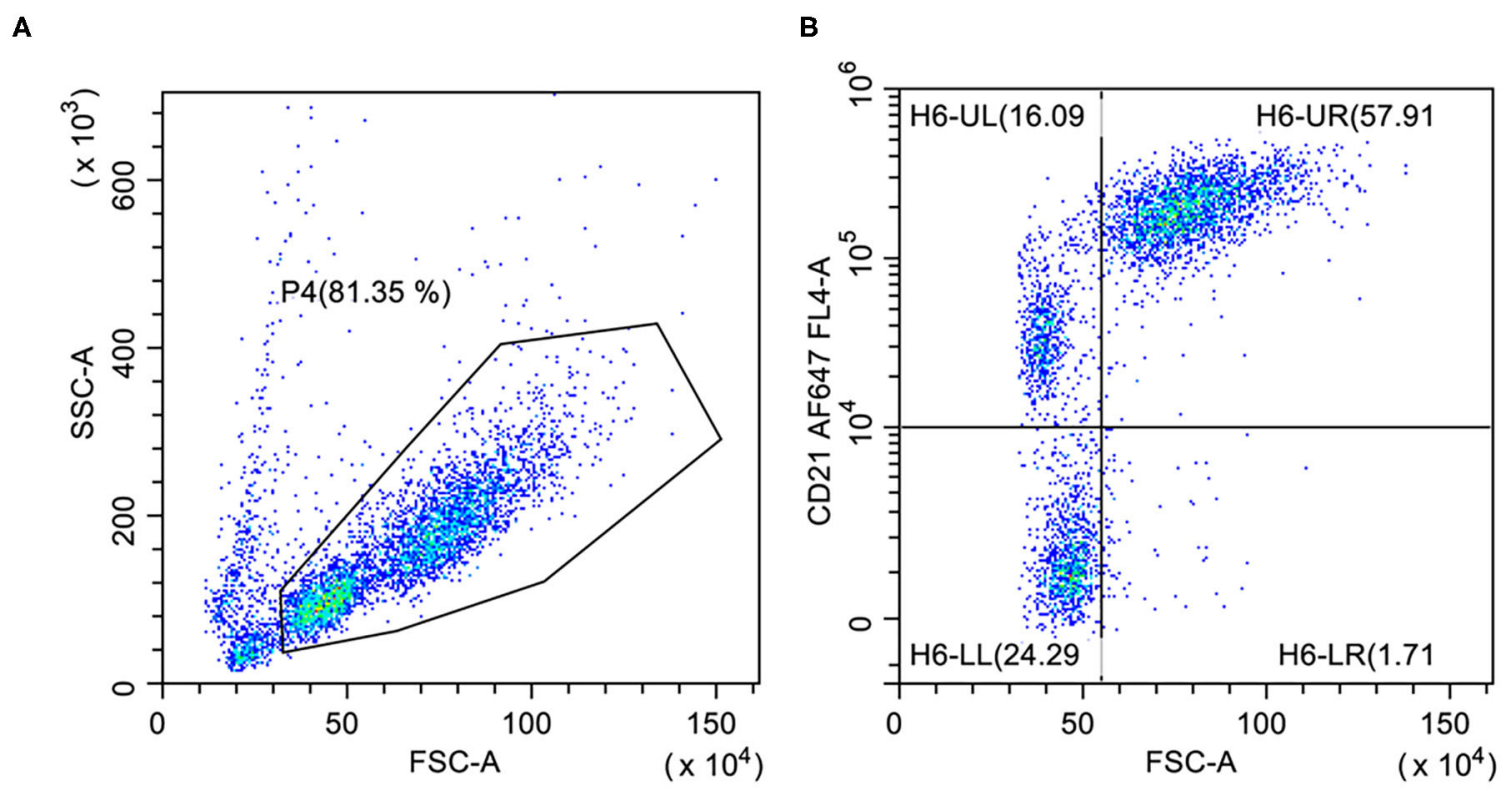
Flow cytometric presentation of a large B-cell lymphoma. **(A)** Forward scatter (FSC) vs. side scatter (SSC) plot after doublet exclusion showing two populations of small- and large-sized cells. **(B)** FSC vs. CD21 plot of P4-gated cells showing higher CD21 expression on large cells (H6-UR) compared with small B-lymphocytes (H6-UL).

The most reliable way to confirm a neoplastic expansion is the identification of one discrete population with aberrant or abnormal antigenic pattern (pseudoclonality) (16). Aberrant co-expression of T-cell or precursor markers (CD34) can sometimes occur (9, 14, 17, 18). Diminished expression of CD79a is also common (17) and CD21+ CD79− or CD21− CD79+ cases are occasionally found (14, 18). However, the probability to detect phenotypic aberrancies is strictly related to the number and type of antibodies included in the panel. Again, the scarce availability of specific antibodies for canine B-cells is the main factor limiting the use of aberrant phenotypes as a major tool to confirm clonality. In the personal experience of the authors, and in agreement with some published papers (11, 19), large BCLs commonly have a higher expression of CD21 if compared to small non-neoplastic B lymphocytes (Figure 2). However, a population with similar size and CD21 intensity was observed in the spleen of healthy dogs (19). This finding suggests that the large size of the cells and the upregulation of CD21 cannot unequivocally differentiate neoplastic and non-neoplastic B-lymphocytes.

FC cannot provide a definitive diagnosis of B-cell lymphoma if there is no evidence of an obvious neoplastic population (i.e., phenotypic aberrances and/or a high percentage of cells with the same phenotype) and in presence of a doubtful phenotype (i.e., absence or co-expression of T- and B-cell markers). In these cases, histopathology and immunohistochemistry remain mandatory. Additionally, PCR for Antigen Receptor Rearrangements (PARR) analysis can help in achieving the correct diagnosis and define lineage allocation (20); however it can be less sensitive and accurate than FC to predict B and T phenotype (10).

## Refining The Diagnosis Of B-Cell Lymphoma Subtypes

Although it may add some useful information to the diagnosis of lymphoma and phenotyping (see below), FC is not currently able to distinguish different types of BCL according to the WHO classification system in dogs. Most BCLs diagnosed in dogs are positive for both CD21 and CD79, while, CD21− CD79+ and CD21+ CD79− lymphomas are less common (14, 18). Expression of T-cell markers in BCLs (lineage infidelity) may occasionally occur (9, 10) but this aberrant expression has not been correlated to any specific subtype. The cell size determined by FC may be of aid in further classifying B-cell neoplasms: as in cytology and histology, DLBCLs have large-sized cells while follicular and diffuse small BCLs are characterized by smaller cells (15). Unfortunately, most BCLs appear as medium to large-sized and the identification of large cells does not rule out subtypes other than DLBCL (15). In this context, the determination of Ki67, a proliferation antigen, can help in discriminating between high and low-grade forms (21). However, in the author's experience, histologically diagnosed MZLs may present variable flow cytometric size (medium to large) and Ki67 values (indicative of both low and high-grade forms). Therefore, it is not possible to differentiate large-sized high-Ki67 MZLs and DLBCLs by flow cytometry alone (15, 22). This flow cytometric presentation is consistent with a late stage of the disease with more aggressive behavior (23) or, as described in humans, it can represent a transformation of MZL in DLBCL. A similar hypothesis has been formulated in dogs (24) and it is supported by the similar molecular profile of MZL and DLBCL in this species (25, 26).

Finally, no differences in the immunophenotype of different subtypes of BCL were detected in one study (15). This limits the current role of FC immunophenotype in the sub-classification of B-cell lyphomas. However, the study included mostly DLBCL, and possible phenotypic differences in other less frequent lymphoma subtype should be further assessed.

## Refining The Prognosis Of B-Cell Lymphoma

FC plays a leading role in defining two major characteristics of lymphomas with possible impact on prognosis: cell lineage (B vs. T) and staging (see below). Other FC features potentially related to prognosis may derive from the evaluation of cell size, the reactivity to some specific prognostic markers, and the evaluation of the residual non-neoplastic cell population.

As previously described FSC values are indicative of small, medium, or large cell lymphomas, although a correlation between the flow cytometric and the morphologic information (cytology and histology) has not been assessed so far. Of greater importance, large flow cytometric cell size has been linked to high-grade lymphoma subtypes (22) and a worse prognosis in BCLs (27). A more specific evaluation of the grade of malignancy is provided by Ki67 and S-phase values that may be determined by FC in the lymph node at diagnosis. Cutoff values of 12.2 and 3.15%, respectively, were proposed to identify high-grade forms (21, 28). Furthermore, the percentage of Ki67 stratifies three prognostic groups in dogs affected by high-grade BCLs: intermediate Ki67 values (20.1–40%) were associated with longer relapse-free interval and survival time (median 428 and 866 days, respectively) compared with low (<20%; median 159 and 42 days) and high values (>40%; median 100 and 173 days) in one study (29).

Recently, additional prognostic markers have been investigated by FC. Low levels of MHC II expression predicted a poor outcome in BCLs in two studies (13, 27). However, this result was not confirmed in a cohort of DLBCLs in a later study (15). This discrepancy is likely due to the possible inclusion of different BCL subtypes in the first studies. High levels of CD25 have been associated with a worse prognosis, resembling human DLBCL (15, 30).

One of the main advantages of FC is the multiparametric approach. Numerous parameters or markers can be evaluated on every single cell at the same time. This feature allows an accurate and sensitive description of different subsets within a population. The residual populations of non-neoplastic T lymphocytes are easily identifiable in BCLs and they appear to play a role in the progression of the neoplasm, thus suggesting an active influence of the immune system on the tumor pathobiology and treatment response. A higher percentage of regulatory T-cells (FoxP3+) has proven to be an independent prognostic factor in BCLs (13) and a recent study showed that higher percentages of CD5+ and CD8+ lymphocytes and lower CD4+/CD8+ ratio at diagnosis are associated with a lower likelihood of progression in dogs with DLBCL treated with chemo-immunotherapy (31).

FC analysis of peripheral blood can be run to calculate the lymphocyte-to-monocyte ratio. This ratio has been reported as an independent prognostic factor and a value <=1.2 at the diagnosis is associated with shorter time to progression and survival in dogs with DLBCL treated with chemoimmunotherapy (32). Similar cutoff (1.43) and results were obtained using a lymphocyte-to-monocyte ratio calculated from hematology reports in another study (18), as also described in human medicine (33, 34).

**Figure 3:**
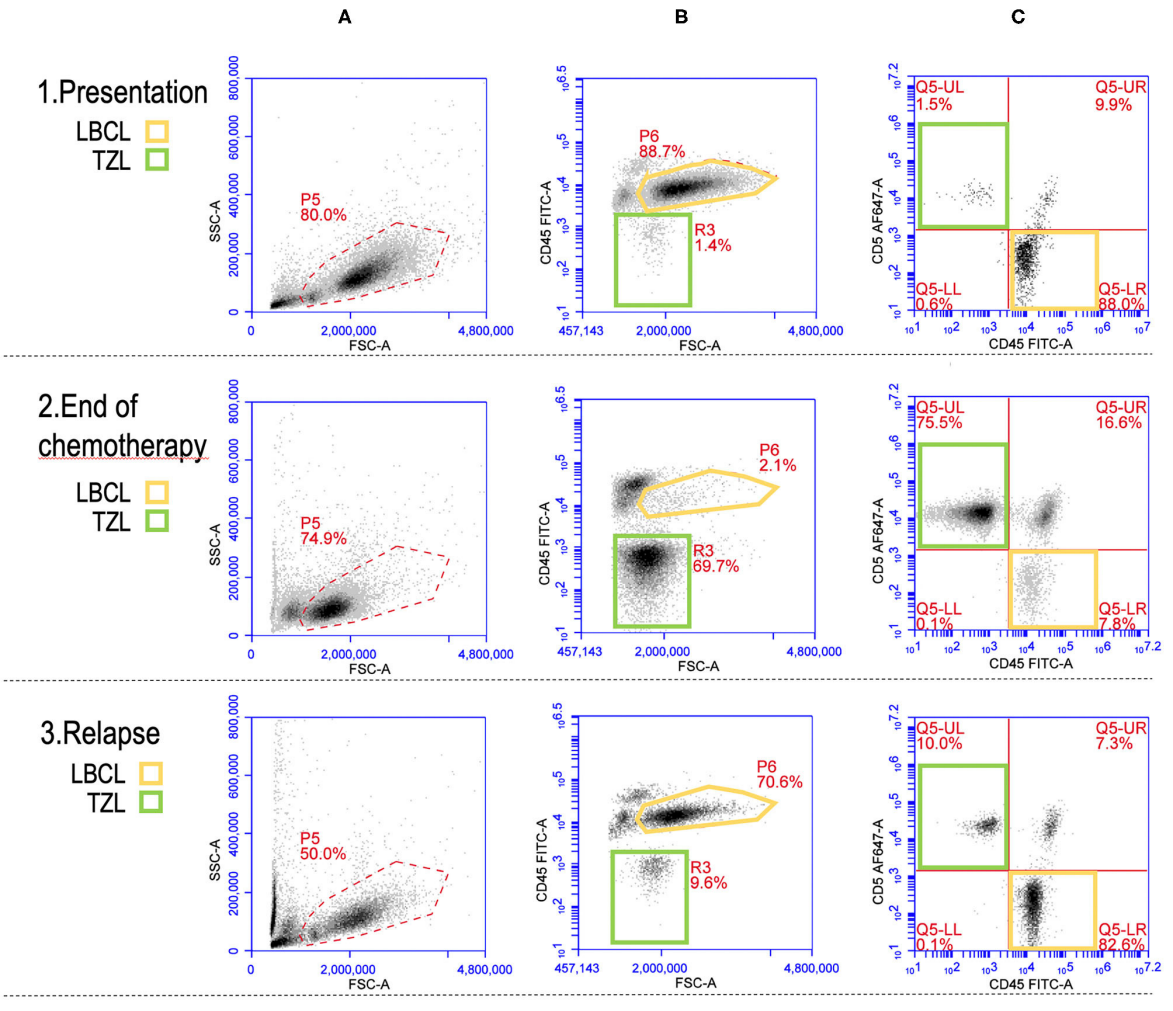
Flow cytometric picture of a large B-cell lymphoma (LBCL) and a concurrent T-zone lymphoma (TZL) at presentation (row 1), end of CHOP chemotherapy (row 2), and relapse of LBCL (row 3). Yellow gates (P6 and Q5-LR): LBCL cells. Green gates (R3 and Q5-UL): TZL cells. **(A)** plots (FSC vs. SSC): “morphologic” presentation of the whole nodal population with gate (P5) activated for the analysis in plots **(B,C)**; a prevalence of large-sized cells (higher FSC) is recognizable at presentation and relapse while small and medium-sized cells are predominant at the end of chemotherapy. **(B)** plots (FSC vs. CD45): CD45 expression according to cells size (FSC); the main population is represented by large CD45+ cells (P6, yellow) at presentation and relapse while it is made of medium CD45− cells (R3, green) at the end of chemotherapy. **(C)** plots (CD45 vs. CD5): most of the events are CD45+CD5− (Q5-LR, yellow) at presentation and relapse while CD45-CD5+ cells (Q5-UL, green) represent the majority of the population at the end of chemotherapy.

Finally, the multiparametric approach of FC is extremely useful also to identify rarer cases of two coexisting neoplasms, such as T-zone lymphoma and BCL (35–37). An example is shown in Figure 3, where both BCL and TZL populations are shown at diagnosis, at the end of CHOP chemotherapeutic protocol, and at relapse of BCL.

## Staging B-Cell Lymphoma

FC is an excellent tool for staging lymphoma (i.e., to identify neoplastic cell infiltration in different tissues), in particular in fluid matrices including peripheral blood (PB) and bone marrow (BM). The major advantage is that it allows examination for populations with the same scatter and immunophenotypic properties of the primary lesion. Good analytical performances have been reported in the quantification of PB and BM infiltration in dogs with large BCLs (38). Given the good accuracy and precision reported and the differences in results provided by operators in the evaluation of smears, FC appears a more reliable method compared to microscope observation (39). A sensitivity of 0.5 and 2% in blood and bone marrow, respectively, and a diagnostic cutoff of 0.56 and 2.45% were identified for flow cytometric detection of neoplastic large B-cells (38). These values depend on the use of the currently available antibody panel, therefore their accuracy would be likely improved with the availability of new specific markers. Anyway, they appear acceptable especially considering the prognostic threshold of 3% for BM infiltration in large BCLs that has been reported in another study (40). In contrast, no PB cutoff values with prognostic significance have been described for large BCLs so far.

The issue of staging BM infiltration is debated. Some oncologists do not consider BM analysis mandatory to accurately define the prognosis. This is probably because of the invasiveness of the procedure and because bone marrow evaluation is not considered necessary in absence of cytopenias, presence of circulating blast cells, or other hematological alterations. However, previous studies on DLBCL demonstrated that PB and BM involvements are not directly correlated and it is not possible to predict BM infiltration out of PB results (23, 40). Furthermore, PB picture is not always predictive of marrow involvement (41, 42), even though dogs with severe BM infiltration often show some hematological alterations (thrombocytopenia, lymphocytosis, circulating neoplastic cells). Thus, both tissues should be included in routine staging to correctly stage the disease.

Regarding other BCL subtypes, a recent paper reported a significant association between PB and BM infiltration and prognosis in dogs affected with nodal MZL. Dogs with <30% PB infiltration showed a significantly longer time to progression while BM infiltration stratified cases in three groups (<1%; 1–20%; > = 20%) with different survival times (43).

## Evaluation Of Minimal Residual Disease

The major role of FC in the follow-up of lymphoma patients is related to the identification of the minimal residual disease (MRD) in PB, BM, and lymph node at the end of the chemotherapeutic protocol. In humans, MRD evaluation plays a key role in the treatment plans of hematologic malignancies and allows individualized tailoring of therapies, in particular for leukemic patients (44). Similar to staging, FC can detect residual neoplastic cells in PB, BM, or lymph node looking for populations with the same characteristics described at diagnosis, and thus it is a useful tool to assess the efficacy of therapy.

A study evaluating MRD in lymph nodes, PB, and BM found only a moderate agreement between FC and PARR at the end of chemotherapy in dogs with DLBCL. Flow cytometric cutoff was set at 1%. PARR was more sensitive than FC in predicting time to relapse but only the combined interpretation of the two methods was predictive of the overall survival (45). One of the reasons for the only moderate concordance is the different sensitivity of the two methods that in turn depends also on the threshold used to define the positive and negative status. In this perspective, a recent study showed a percentage >0.5% of large CD21+ cells in lymph node to be predictive of a shorter time to relapse in dogs with DLBCL in complete clinical remission (46). To the best of our knowledge, no other studies have been published on the prognostic significance of MRD in PB and BM so far.

## Future Perspectives

The development of new specific antibodies suitable for FC and targeting more B-cell antigens may be useful to improve the accuracy of the diagnosis of BCL and improve prognostic abilities. In particular, specific antibodies recognizing plasma cells (CD38) or immature B cells (CD20, CD19, CD10) labeled with different fluorochromes are needed to be included in the diagnostic panels.

The wide range of possible applications of FC makes it an important tool for future developments in the field of canine lymphoma. FC can contribute to the discovery of new therapeutic targets and identification of new drugs with the ultimate goal of more individualized therapies. Some examples include studies focusing on specific markers such as PD-1 / PD-L1 (47, 48), c-kit (49), or characterizing cell behavior such as the proliferative and /or apoptotic response to different molecules (50–56), or aimed to produce and characterize of new monoclonal antibodies (57, 58).

The diagnostic usefulness of flow cytometric analysis in dogs affected by lymphoma is increasing. In this review, we summarized recently reported flow cytometric indexes and features with clinical significance for canine B-cell lymphomas. To confirm and validate the diagnostic and prognostic role of these parameters, larger multicentric prospective studies are warranted. Collectively, they represent important milestones in the exploration of the full power of flow cytometry in veterinary oncology. They lay the foundation to understand why similar lymphomas behave differently and to improve the ability to differentiate prognosis among B-cell neoplasms and within a single lymphoma subtype. Currently, flow cytometry has the highest diagnostic value if combined with other morphologic techniques such as cytology and/or histology. Its potential will be expanded in the future but we believe the right way is to aim for the result of fully integrated multidisciplinary (flow cytometry, histology, cytology, genetic, molecular) lymphoma diagnosis.

## Author Contributions

All authors listed have made a substantial, direct and intellectual contribution to the work, and approved it for publication.

## Conflict of Interest

The authors declare that the research was conducted in the absence of any commercial or financial relationships that could be construed as a potential conflict of interest.
